# Diagnostic odyssey in amyotrophic lateral sclerosis: diagnostic criteria and reality

**DOI:** 10.1007/s10072-023-06997-1

**Published:** 2023-08-08

**Authors:** Stanisław Maksymowicz, Tomasz Siwek

**Affiliations:** 1 Department of Psychology and Sociology of Health and Public Health, School of Public Health, Collegium Medicum of the University of Warmia and Mazury, Warszawska 30 Street, 10-082, Olsztyn, Poland; 2 Department of Neurology, Faculty of Health Sciences, Collegium Medicum of the University of Warmia and Mazury, Olsztyn, Poland; 3 Department of Neurology, University Clinical Hospital, Olsztyn, Poland

**Keywords:** ALS, Diagnosis, Communication, Odyssey, Diagnostic criteria

## Abstract

**Background:**

Diagnosing a rare disease, such as amyotrophic lateral sclerosis, is a major challenge for physicians and patients. Despite detailed diagnostic criteria, this process often does not proceed as it should, exacerbating the problems of patients. In the following study, we show how the process, which in medical sciences has been called the “diagnostic odyssey”, proceeds and how it affects patients.

**Materials and methods:**

Participants were recruited via a neurology clinic. Twenty-four patients with the diagnosed disease were interviewed using in-depth interviews and an author questionnaire: 9 females and 15 males ages ranging from 30–39 to 60–69.

**Results:**

The median time from 1st symptoms to diagnosis was almost 12 months and mean almost 20 months (min. 3, max 106). Only 5 patients waited less than 6 months for being diagnosed. Over 80% of patients received an alternative diagnosis on the first attempt.

**Conclusion:**

ALS is a fast-paced fatal disease, which requires immediate action to slow down the course of the disease and improve patients’ quality of life. However, in many cases, the disease is diagnosed too late. It also happens that a wrong diagnosis causes inaccurate treatment, which accelerates the development of ALS. For this reason, it is necessary to expand the clinical and communication competences of medical personnel already at the stage of medical studies. In addition, the diagnostic criteria should highlight the common problem with diagnosing ALS.

## Introduction

Amyotrophic lateral sclerosis (ALS) is a rare, incurable, and fatal neurodegenerative disease characterized by progressive muscular paralysis. This devastating disease occupies both central and peripheral motor neurons [1], rapidly bringing the patient to disability. Median survival amounts from 37 to 49 months [2]. The prevalence of ALS ranges from about 1/100,000 to even 8/100,000 in some regions [3, 4].

Due to the unfavorable nature of the diagnosis and ALS rapid course, prompt diagnosis and immediate treatment are of particular importance. Unfortunately, in many cases, this does not happen, and patients, instead of receiving quick help, wait for a diagnosis for months.

The study aimed to find out how the diagnosis of amyotrophic lateral sclerosis was carried out, including how long patients waited for the final diagnosis from the first symptoms they observed [5] and what diseases were diagnosed and even treated before the final diagnosis of ALS. Finally, we have also proposed recommendations for clinicians that can help improve the diagnostic process by taking into account patients’ perspectives.

## Material and methods

The study approved by the Scientific Research Ethics Committee of the University of Warmia and Mazury in Olsztyn (decision no. 5/2018) was conducted using the PAPI method (pen-and-paper personal interviews) between February and June 2018. The study was based on the standardized original questionnaire containing 30 closed questions (demographic, diagnostic path, and opinions on the diagnosis), as well as 5 open questions (related to the patient’s experience in each area). The questionnaire translated from the Polish original is included in our previous paper describing breaking bad news [6]. The subjects of the study were patients of a private neurology clinic in Poland with diagnosed ALS based on El Escorial standards [7]. Important rules qualifying patients were the ability to communicate—even if with the help of a carer—and good mental health.

Thirty patients were qualified for the study, but ultimately results were collected from 26 people. Two cases were rejected due to the lack of full answers. Finally, 24 cases were used in the analysis which is a significant outcome given the rarity of the disease.

**Fig. 1:**
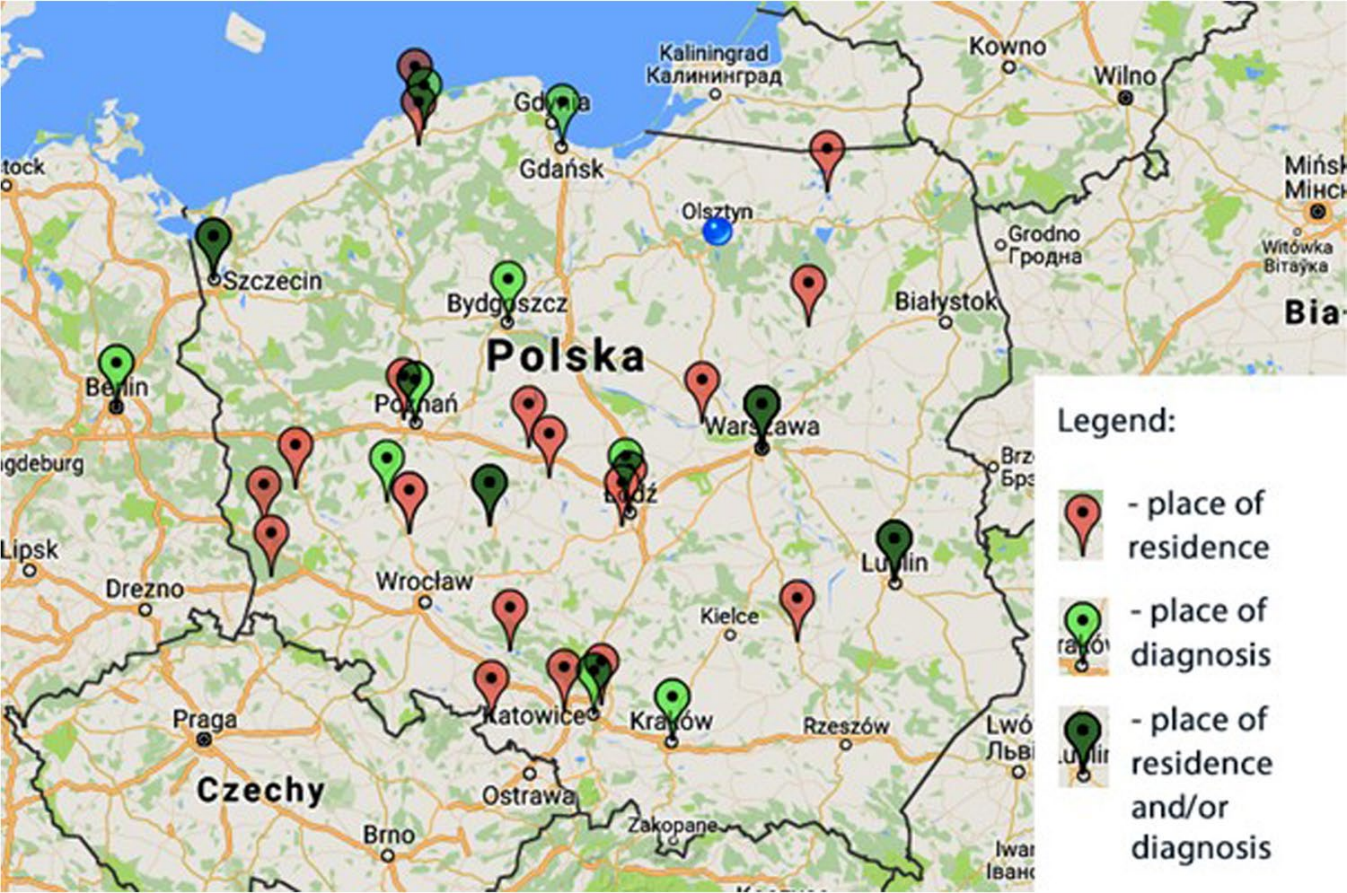
Map of a place of residence and diagnosis (map: maps.google.com)

The obtained research group (Table 1) was demographically non-homogeneous: age was spread over 3 cohorts: 40–49 (33.3%), 50–59 (37.5%), and 60–69 years (25%). The distribution of sex (15 males and 9 females) corresponds to the distribution occurring in ALS and is characterized by the predominance of men with a 2:1 male-to-female gender ratio [8, 9]. The distribution of the sample around the “place of residence” variable was also relatively large, with a small advantage of the big city (over 250 t.) Respondents came also from different regions of Poland. The place of diagnosis is also varied as shown in Fig. 1.

**Table 1:** Patients characteristics

Demographic data	*N* = 24
Sex	
Male Female	15 (62.5%) 9 (37.5%)
Age	
30–39 40–49 50–59 60–69	1 (4.2%) 8 (33.3%) 9 (37.5%) 6 (25%)
Place of residence	
Village City up to 50 t City up to 100 t City up to 250 t City over 250 t	3 (12.5%) 5 (20.8%) 4 (16.7%) 5 (16.7%) 8 (33.3%)
Education	
Elementary Secondary Higher	3 (12.5%) 10 (41.7%) 11 (45.8%)
Onset	
Limb Bulbar	16 (66.7%) 8 (33.3%)

## Results

**Fig. 2:**
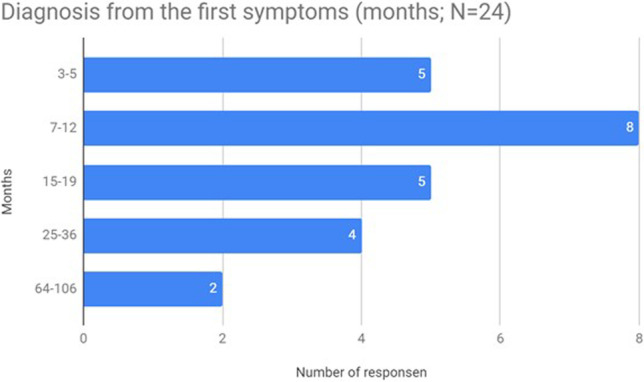
Time from first symptoms to ALS diagnosis

The median time from 1st symptoms to diagnosis was 12 months, and the medium time was 19.75 months (min 3, max 106, IQR: 15, Table 2). Only 5 patients waited less than 6 months for being diagnosed (Fig. 2). The largest value (106 months) refers to a patient with a chronic form of ALS and has ignored the symptoms himself. However, the remaining values were patients who received a late diagnosis due to a process called “diagnostic odyssey” which means that patients were diagnosed and treated for a long time by different specialists in different centers, unable to obtain a reliable diagnosis [5, 10].

**Table 2:** Diagnosis time (months)

*N* valid	24
Mean	19.75
Median	12.00
Std. deviation	22.828
Range	103.00
Minimum	3.00
Maximum	106.00
Quartiles	Q1: 7 Q2: 12 Q3: 22
Interquartile range (IQR)	15

One of the variables that had a significant impact on the prolonged time of diagnosis was the type of onset of ALS (*p* = 0.032) and patients with limb onset waited longer for diagnosis. Among the demographic factors that statistically significantly affected the length of diagnosis (delayed diagnosis) in the study group were male gender (*p* = 0.034) and higher education (*p* = 0.000). In the case of the group we analyzed, age had no significant effect. It seems, however, that this study group is too small and too little diversified, especially in terms of education to allow a broader conclusion in terms of demography.

**Fig. 3:**
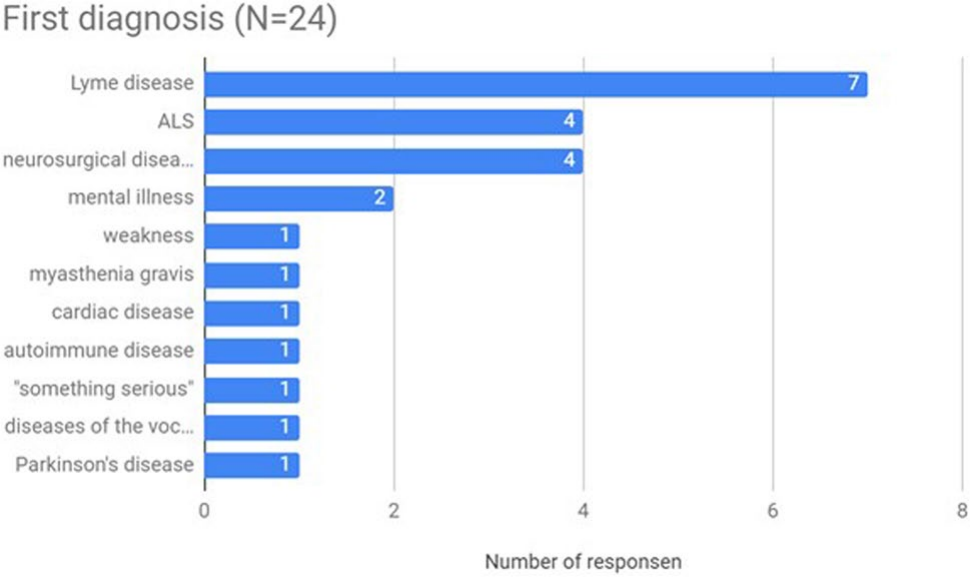
First diagnosis based on symptoms

Most of the patients (83%) received an alternative diagnosis after the first symptoms (Fig. 3, Table 2). Before a proper diagnosis, most of them were diagnosed with Lyme disease (*n* = 7), neurosurgical diseases (spinal injury, etc., *n* = 4), and mental disorders (neurosis, *n* = 2). Other diseases diagnosed were autoimmune, myasthenia gravis, cardiac, Parkinson’s, vocal cords disease, and in two cases “other problems”—overall weakness and “something serious”. ALS as the first diagnosis was indicated by 4 patients. Moreover, 8 patients (33%) admitted that they diagnosed themselves before visiting a doctor. Despite this, the diagnosis process was often carried out by the doctor without taking into account patients’ self-diagnosis.

It also happened that the 2nd or even the 3rd diagnosis was made incorrectly before the final diagnosis was made or the diagnosis of ALS was changed to a different health problem (Table 3).

**Table 3:** Diagnostic odyssey in the study group (“-” means, that ALS was finally diagnosed, *N* = 24)

Diagnosis 1	Diagnosis 2	Diagnosis 3	First symptoms (perceived by the patient)	Time (from 1st symptoms to diagnosis; months)	Treatment attempts
Lyme disease	–	–	Speech deterioration	10	–
Lyme disease	–	–	Unsteady gait	7	–
Autoimmune disease	–	–	Hand weakness	15	–
Weakness	–	–	Choking	27	Yes (condition deteriorated)
Myasthenia gravis	–	–	Speech deterioration	12	Yes (myasthenia treated for half a year, the condition deteriorated)
Lyme disease	Paraneoplastic disease	–	Hand weakness, pain	5	–
Neurosurgical diseases	–	–	Hand weakness	19	
Mental illness	Lyme disease	–	Tingling on the face	12	Yes (1 year of psychiatric treatment, worsened condition)
Lyme disease	Neurosurgical diseases	–	Hand and arm cramps	23	Yes (spine rehabilitation without improvement effects)
Cardiac disease	–	–	Shortness of breath	19	Yes (stents placed)
Neurosurgical diseases	–	–	Hand weakness	9	–
Mental illness	Lyme disease	–	Loss of balance, trouble walking	9	–
“Something serious”	–	–	Left foot drop	16	–
The disease of the vocal cords	Nerve inflammation	Stroke	Deterioration of speech, weakness of the hands	64	Yes (anti–inflammatory injections, hospitalization for suspected stroke)
Lyme disease	Parkinson’s disease	Cancer	Hand tremor	36	–
ALS	Neuropathy	–	Unexpected leg cramps, weight loss	25	–
Neurosurgical diseases	Neuropathy	–	Back, neck, and shoulder pain	31	Yes (massages that worsened condition)
Lyme disease	–	–	Hand weakness	5	Yes (1.5 years of ilads antibiotic therapy, worsened condition)
Parkinson’s disease	–	–	Hand weakness	10	–
Lyme disease	Neurosurgical diseases	–	Shortness of breath and choking	7	The patient was referred for spinal surgery, which ultimately did not happen
Neurosurgical diseases	–	–	Stumbling, difficulty climbing stairs	19	Yes (orthopedic treatment, collar)
ALS			Muscle fasciculations	106	–
ALS			Muscle fasciculations	5	–
ALS			Respiratory symptoms (dyspnea, choking)	3	–

The result of an incorrect diagnosis was another observed problem: over 41% (10 patients) of patients were treated for diseases they did not have, including antibiotic therapies for Lyme disease, medicines for myasthenia, and psychotherapy. As the respondents emphasized, the treatment only slowed the treatment of ALS and even led to a significant deterioration of health (Table 3).

## Discussion

ALS is a rapidly progressing disease, and because of that, patients often indicated that they would like to learn about it as possible, even at the first suspicions by the doctor [6]. This would allow them to take immediate action that may delay the disease’s progression.

However, from a clinical point of view, the process of investigation from suspicion to diagnosis in medicine always requires diligence and entails liability for the consequences of an incorrect diagnosis. It includes history taking, physical examination, additional tests, and often consultations. In the case of chronic and rare diseases, the non-simultaneous appearance of symptoms and their potential sequence may be crucial for the time from their onset to the correct diagnosis. Also, the neurophysiological signs in EMG and their comparison to the diagnostic criteria of ALS are very important to diagnosis and level of evidence [11, 12]. However, this can be confusing as ALS is a disease with symptoms that connect with many different health problems, especially Lyme disease, neuropathy, myasthenia gravis, neurosis, and spine injuries [13].

On top of that, there is also the perspective of a doctor—a person who is the messenger of an unfavorable diagnosis. Information about diseases with a poor prognosis and without the prospect of effective treatment is provided to patients with extreme caution—usually after thorough verification and obtaining certainty about its truthfulness. Often, the mere suspicion of a disease by a doctor raises so much concern that diagnosis is made under confidential conditions until the most accurate results and verification are obtained. These practices are designed to protect the patient from exposure to unjustified fears for health and life—but are they ethical and effective [14]? As we have already indicated, most of the patients we surveyed would like to hear a diagnosis even at the first suspicion.

However, even raising suspicion can be difficult. In ALS, neurological symptoms start slowly, with no pain symptoms, which often put anxiety to sleep. The first to appear are motor deficits, muscle atrophy, or speech and swallowing disorders. This leads to starting diagnostics with potentially different specialists: neurologists, neurosurgeons, phoniatrist, and speech therapists. This is a potential factor that extends the patient’s journey to a neurological or neuromuscular clinic [5, 15].

## Conclusions

In our study, as well as based on the literature, it can be concluded that the process of the diagnostic odyssey in ALS, but also in other chronic and rare diseases, is a reality faced by both clinicians and their patients on a daily basis. The causes of this problem lie both in the sphere of the patient’s perception, the knowledge and diagnostic abilities of experts, and the way the healthcare system functions.

Factors that may delay the diagnosis in ALS include above all:

- Time from 1st symptoms to reasonable suspicion and referral for neurophysiological diagnostics
- Waiting time for tests and results
- Waiting time for follow-up visits
- Differential diagnosis period with low confirmation criteria (waiting for the fulfillment of El Escorial/Awai/Gold Coast diagnostic criteria)
- Delay by diagnosing and treating the “dead ends” of differential tests
- Waiting time to reference to diagnostic centers
- The need for the patient to verify the truth of “bad news” with other specialists

How can this problem be addressed? The basic answer is education at the stage of medical studies, which should be focused on careful medical history taking [16], also from the side of medical communication, self-awareness of one’s limitations and routine [17]. In addition, the diagnostic criteria should highlight the common problem with diagnosing ALS. The diagnostic criteria of ALS evolve from useful mostly in experimental trials to more helpful for clinical practice [7]. Those changes and of course wide knowledge of current criteria by neurologists may minimize the delay of diagnosis.

## Data Availability

All raw data in anonymized form are available upon notification to the corresponding author.
